# Transcriptome Analysis Identifies Accumulation of Natural Killer Cells with Enhanced Lymphotoxin-β Expression during Glioblastoma Progression

**DOI:** 10.3390/cancers14194915

**Published:** 2022-10-07

**Authors:** Gianni Monaco, Ashkan Khavaran, Adrià Dalmau Gasull, Jonathan Cahueau, Martin Diebold, Chintan Chhatbar, Mirco Friedrich, Dieter Henrik Heiland, Roman Sankowski

**Affiliations:** 1 Institute of Neuropathology, Faculty of Medicine, University of Freiburg, 79106 Freiburg, Germany; 2 Single-Cell Omics Platform Freiburg, Faculty of Medicine, University of Freiburg, 79106 Freiburg, Germany; 3 DKTK Clinical Cooperation Unit Neuroimmunology and Brain Tumor Immunology, German Cancer Research Center (DKFZ), 69120 Heidelberg, Germany; 4 Department of Neurosurgery, Faculty of Medicine, University of Freiburg, 79106 Freiburg, Germany; 5 Microenvironment and Immunology Research Laboratory, Medical Center-University of Freiburg, 79106 Freiburg, Germany; 6 Comprehensive Cancer Center Freiburg (CCCF), Faculty of Medicine and Medical Center-University of Freiburg, 79106 Freiburg, Germany; 7 German Cancer Consortium (DKTK), Partner Site Freiburg, 79106 Freiburg, Germany

**Keywords:** glioblastoma, GL261, immunotherapy, lymphotoxin, natural killer cell, mesenchymal transcriptional subtype, immune cell crosstalk

## Abstract

**Simple Summary:**

Natural killer (NK) cells play a pivotal role in anti-cancer immunity. We conducted an integrated analysis of single-cell RNA-sequencing datasets of malignant brain tumor-associated immune cells from a mouse glioma model and human newly diagnosed and recurrent glioblastomas. We found a robust increase in NK cells during brain tumor production. The cells showed a gene expression phenotype associated with NK cell dysfunction. Notably, the cytokine Lymphotoxin-β (LTB) was highly expressed in these cells. LTB receptors are found on myeloid and structural cells. In summary, we characterize a dysfunctional NK cell phenotype in advanced glioma.

**Abstract:**

Glioblastomas are the most common primary brain tumors. Despite extensive clinical and molecular insights into these tumors, the prognosis remains dismal. While targeted immunotherapies have shown remarkable success across different non-brain tumor entities, they failed to show efficacy in glioblastomas. These failures prompted the field to reassess the idiosyncrasies of the glioblastoma microenvironment. Several high-dimensional single-cell RNA sequencing studies generated remarkable findings about glioblastoma-associated immune cells. To build on the collective strength of these studies, we integrated several murine and human datasets that profiled glioblastoma-associated immune cells at different time points. We integrated these datasets and utilized state-of-the-art algorithms to investigate them in a hypothesis-free, purely exploratory approach. We identified a robust accumulation of a natural killer cell subset that was characterized by a downregulation of activation-associated genes with a concomitant upregulation of apoptosis genes. In both species, we found a robust upregulation of the Lymphotoxin-β gene, a cytokine from the TNF superfamily and a key factor for the development of adaptive immunity. Further validation analyses uncovered a correlation of lymphotoxin signaling with mesenchymal-like glioblastoma regions in situ and in TCGA and CGGA glioblastoma cohorts. In summary, we identify lymphotoxin signaling as a potential therapeutic target in glioblastoma-associated natural killer cells.

## 1. Introduction

Glioblastomas are aggressive primary brain tumors with a median survival of 14.6 months despite standard-of-care treatment [1]. Evidence from single-cell RNA sequencing studies suggests that glioblastomas and other gliomas arise from a transformation event of glial progenitor cells [2,3]. In line with this origin, glioblastoma cells express astrocyte-like (AC)-like, neural progenitor cell (NPC)-like, oligodendrocyte progenitor (OPC)-like, mesenchymal (MES)-like and other transcriptional programs [2,4,5]. These states are concomitantly found within the same tumor, affecting different functional and prognostic aspects of glioblastoma biology. Recent evidence suggests that neuroectodermal-like tumor cells, i.e., NPC-, OPC- and AC-like cells, engage in crosstalk with resident brain cells, driving diffuse tumor infiltration, while MES-like regions are found at the tumor core [6]. Different modes of crosstalk between neoplastic and resident cells have been described, including microtubules, synapse formation and secretion of soluble factors [6,7,8,9]. MES-like regions are characterized by immune cell infiltrates, hypoxia and genomic instability [2,10]. Integration of molecular and clinical data has associated MES-like regions with aggressive clinical behavior [11,12]. The influences of chronic inflammation and hypoxia-driven genomic instability and epigenomic reprogramming on tumor evolution within MES-like regions are an area of active research [6,13]. Herein, immune cells, a major component of the glioblastoma microenvironment [14], are of particular interest as vectors of targeted immunotherapies.

Several single-cell RNA sequencing studies have examined the myeloid and T lymphoid compartments that represent the most common immune cells in glioblastomas. The complex interactions between T cells, myeloid cells, non-neoplastic astrocytes and glioma cells have recently been highlighted as drivers of the immunosuppressive glioblastoma microenvironment [15,16,17,18,19]. It was shown that brain resident microglia are gradually replaced during tumor progression in humans and mice [20,21]. While glioma-associated macrophages are largely characterized by cytokine/chemokine, antigen presentation, angiogenesis and hypoxia-associated signaling [20,21,22,23], glioma-associated dendritic cells (DCs) show a remarkable heterogeneity with hallmarks of impaired function [20,24]. T cells display the molecular hallmarks of anergy and exhaustion [25]. A recent study found an unexpected expression of an NK cell-like transcription program in glioblastoma-associated cytotoxic T cells highlighting commonalities between adaptive and innate immunity [26]. Conversely, the functional parallels between NK cells and cytotoxic T cells are well-established [27].

NK cells are innate lymphoid cells with a central role in anticancer and antiviral immunity [28]. Although they are relatively sparse in glioblastomas [26], several NK-cells targeting tumor immune evasion mechanisms have been identified. Different mechanisms are involved, including integrin and NKG2D ligand binding [29,30,31]. Here, we show that glioma progression is associated with an NK cell transcriptional phenotype characterized by reduced activation, an upregulation of apoptosis genes and enhanced lymphotoxin signaling.

We have integrated single-cell RNA sequencing datasets from GL261 syngeneic glioma models that were obtained 14, 21 and 28 days after injection [20,21,32]. The NK cells showed a robust correlation with the time lapsed after tumor injection. The pseudotime analysis revealed a downregulation of interferon genes and an upregulation of Lymphotoxin-β (LTB), a member of the TNF superfamily involved in the development of adaptive immunity and inflammation [33]. LTB is a ligand to Lymphotoxin-β receptors (encoded by the LTBR gene). LTB is expressed by activated T, B and NK cells as well as innate lymphoid cells, while LTBR is expressed on stromal and myeloid cells [33]. Crucially, LTB–LTBR signaling is required for NK cell development [34]. The connection between LTBR signaling and malignancies is well-established [35]. The gene regulatory network and pathway enrichment analysis showed a downregulation of interferon- and an upregulation of apoptosis-associated genes. We confirmed these findings in humans showing that LTB gene expression was spatially correlated with the NK cell exhaustion marker killer cell lectin-like receptor G1 (KLRG1) [36,37,38]. Mining of the Cancer Genome Atlas Consortium (TCGA) and the Chinese Glioma Genome Atlas (CGGA) glioblastoma cohorts showed enhanced LTB expression in MES-like glioblastomas and an association of LTB expression levels with overall survival. In summary, a crosstalk between the lymphoid, tumor and myeloid cells within a glioblastoma drives the chronic inflammation associated with reduced NK cell function.

## 2. Materials and Methods

### 2.1. Data Processing and Analysis

The count data were downloaded from the indicated sources and loaded into R using the Read10x function of the Seurat v4 algorithm [39]. Doublet exclusion was done using the scDblFinder R package v1.10.0 (https://bioconductor.org/packages/release/bioc/html/scDblFinder.html accessed on 15 August 2022). The CITE-Seq mouse dataset from [20] was used as a reference for the multimodal reference mapping workflow specified by the following vignette: https://satijalab.org/seurat/articles/multimodal_reference_mapping.html accessed on 15 August 2022. The remaining mouse datasets were integrated using this workflow with default parameters. The human dataset was integrated using the harmony package with default parameters [40].

The NK cell counts were obtained by assessing the relative abundance of NK cells across all cells and biological replicates. Pearson’s correlation analysis was conducted on each cell type using the stat_cor function of the ggpubr v0.4.0 (https://cran.r-project.org/web/packages/ggpubr/index.html accessed on 15 August 2022).

The latent factors underlying the changes in NK cells were identified using the MOFA+ algorithm [41]. The “integration of a time-course single-cell RNA-seq dataset” workflow was used with default parameters as specified under the following vignette: https://raw.githack.com/bioFAM/MOFA2_tutorials/master/R_tutorials/scRNA_gastrulation.html accessed on 15 August 2022.

The pathway enrichment analysis was conducted using the enrichR web interface [42]. Gene lists from the factors identified using MOFA+ were individually loaded into the website, and the top factors were visualized.

The enrichment of time points in each cluster was assessed using the hypergeometric function with Benjamini–Hochberg adjustment for multiple testing.

The pseudotime analysis was conducted using the StemID2 functionality of RaceID v0.2.6 and FateID v0.2.2. [43,44]. The workflow was run following the vignette specified under: https://cran.r-project.org/web/packages/RaceID/vignettes/RaceID.html accessed on 15 August 2022.

The cell–cell interaction analysis was run using default parameters as specified by the developers. First, human orthologues of the mouse genes were identified using the biomaRt v2.52.0. Then, the counts and relevant metadata were exported, and the analysis was run in the command line as specified in the readme file under: https://github.com/ventolab/CellphoneDB accessed on 15 August 2022.

The transcription factor analysis was conducted using the SCENIC R package [45] using default parameters following the vignette specified under: https://github.com/aertslab/SCENIC/blob/master/vignettes/SCENIC_Running.Rmd accessed on 15 August 2022.

The gene ontology enrichment analysis was conducted using the clusterProfiler v4.4.4. R package [46]. First, the gene names were converted into ENTREZ using the bitr function. Then, the enrichGO function was run.

The spatial co-expression analysis was conducted using the functionality of the SPATA2 v0.1.0. R package. Herein, normalized transcript counts were correlated across 10X Visium spots. Similarly, the spatially weighted correlation analysis of previously defined glioblastoma gene expression signatures with the LTB and LTBR genes was conducted as previously described [10].

Count tables containing the fragments per kilobase of a transcript per million fragments mapped (FPKM) the values of the TCGA and CGGA datasets, and the sample annotations were downloaded from the following URLs: https://www.proteinatlas.org/about/download accessed on 15 August 2022 and http://cgga.org.cn/download.jsp (mRNAseq_325) accessed on 15 August 2022, respectively. The datasets were subset for glioblastoma (“GBM”) and IDH wildtype (“IDHwt”/”Wildtype”). For survival analysis, patients above 20 were considered. The code for survival analysis was modified from: http://www.cgga.org.cn/analyse/RNA-data-survival-result.jsp accessed on 15 August 2022.

### 2.2. Visualization of the Data

The functionalities of the indicated R packages were used where possible. Otherwise, the tidyverse v1.3.2. and ComplexHeatmap v2.12.1. R packages were used [47,48]. The code for the Kaplan–Meier visualization was obtained from: http://www.cgga.org.cn/tools/plot_survival.jsp accessed on 15 August 2022.

Human Protein Atlas image selection and processing: Images regions were obtained from https://www.proteinatlas.org/ENSG00000111321-LTBR/pathology/glioma#img accessed on 15 August 2022. The images were processed in Photoshop 2022 with selective sharpening and increases in vibrancy, brightness and contrast.

## 3. Results

### 3.1. The Mouse Glioma Model GL261 Shows a Diverse Immune Cell Compartment with Changes throughout Tumor Progression

**Figure 1:**
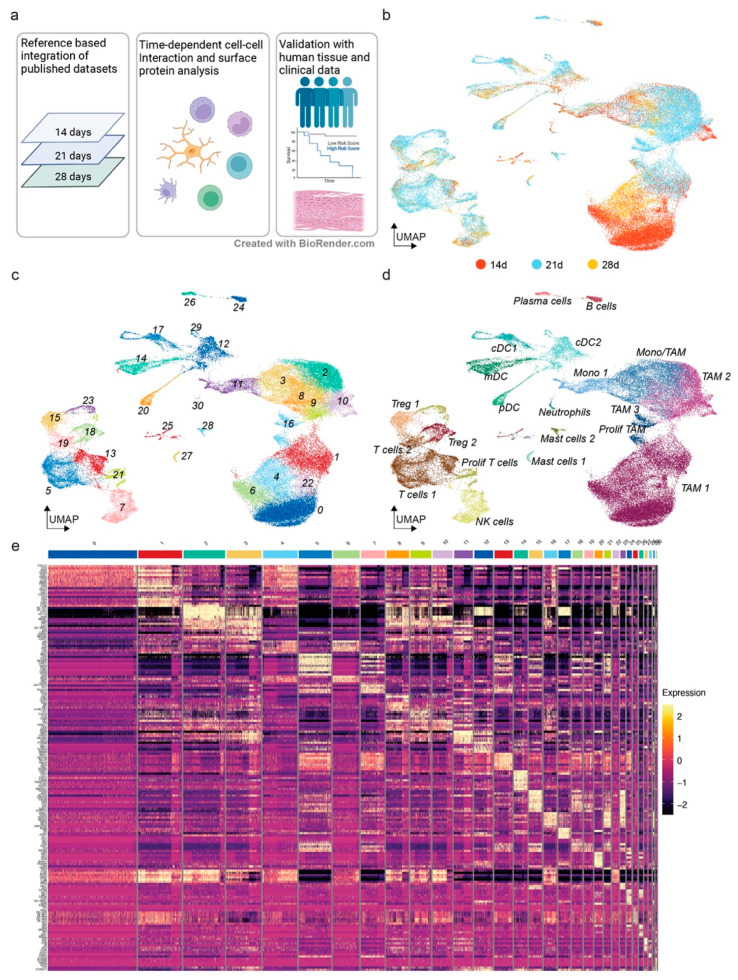
Integration of three GL261 murine orthotopic glioma single-cell RNA-sequencing datasets to analyze tumor progression. (**a**) Schematic illustration of the workflow of the present study. (**b**) UMAP visualization of the integrated dataset (n = 61,814) color-coded by time point. (**c**) UMAP visualization of the integrated dataset color-coded by cluster. (**d**) UMAP visualization of the integrated dataset color-coded by cell type. (**e**) Heatmap visualization of the top 10 marker genes per cluster. The color coding of the respective clusters is consistent with panel c. The color scale indicates the Pearson’s residuals from a negative binomial regression.

**Figure 2:**
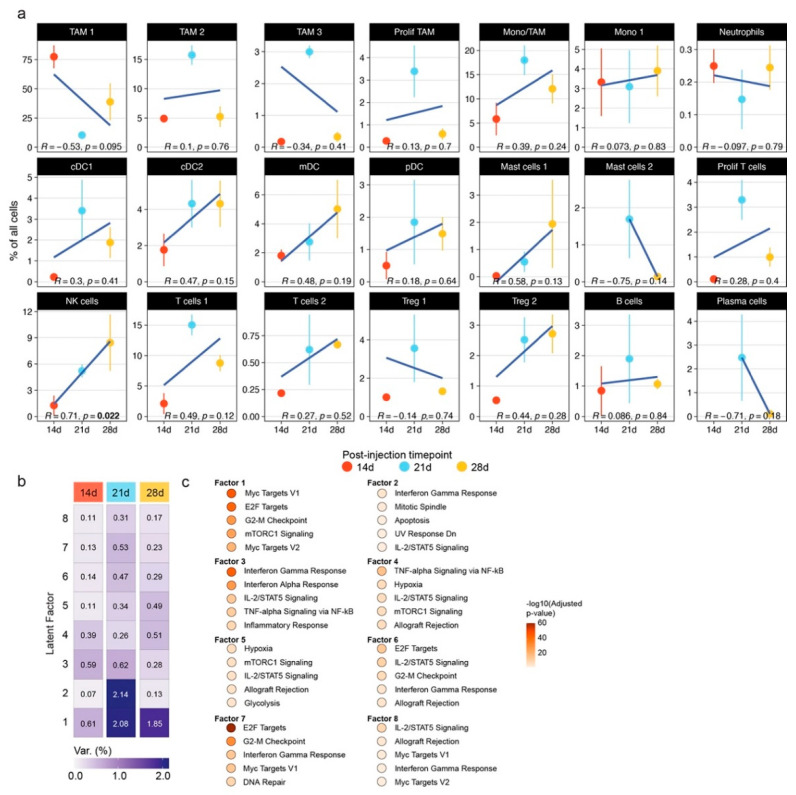
Cell type dynamics during GL261 murine orthotopic glioma progression and transcriptional changes in NK cells. (**a**) Dot line plot visualization of the relative abundances of the indicated cell types as a percent of the total number of immune cells for each time point. The error bar indicates the s.e.m. The cell labels are adapted from the 21 d p.i. time point dataset [20]. At the bottom of each plot, the Pearson’s correlation coefficient and two-sided *p*-value are indicated. (**b**) Heatmap visualization of latent factors determined by MOFA+ analysis. The color scale indicates the percentage of variance explained. For enhanced readability, the respective values are indicated in each cell. (**c**) The enrichR-based pathway enrichment analysis of MSigDB Hallmark 2020 pathways. The color of the dots indicates the -log10-transformed adjusted *p*-values of the enrichment.

We set out to study the longitudinal immune composition of GL261 syngeneic gliomas and validate our findings in human glioblastomas (Figure 1a). In recent years, several studies have profiled the immune compartments in these mice [17,20,21,32,49]. Interestingly, three recent studies with comparatively large immune cell amounts have collected glioma tissues for single-cell RNA sequencing after 14, 21 and 28 days (14 d, 21 d and 28 d) with minor adjustments of the respective protocols [20,21,32]. We utilized the Seurat single-cell RNA-sequencing analysis workflow with reference-based mapping of the datasets from the three abovementioned time points onto a reference dataset containing single-cell transcript and epitope information [39]. We transferred the cell type labels of the reference dataset to be consistent with the integrated dataset [20]. Visual inspection of the UMAP embedding of the integrated dataset confirmed the mixing of the datasets (Figure 1b). Graph-based clustering showed the presence of different cell states in most of the identified cell types [50] (Figure 1c,d). Cluster marker genes were consistent with the published cell type-enriched genes, including the homeostatic microglia clusters 0, 1, 4, 6 and 22 (*P2ry12* and *Hexb*), tumor-associated microglia clusters 2, 8, 9, 10 (*Cd74* and *Apoe*), monocyte and macrophage clusters 3, 8, 11 (*Ly6c2* and *Tgfbi*), T cell clusters 5, 13, 18, 19, 21 (*Cd3d* and *Trbc2*) and NK cluster 7 (*Ncr1* and *Klra4*) (Figure 1e). In order to assess changes in the cell type composition between 14 d and 28 d after GL261 cell inoculation, we conducted a linear regression analysis for the percentages of the respective cell types over the three post-inoculation (p.i.) time points (Figure 2a). Some cell types showed shifts in abundance without reaching a statistical significance of 0.05, e.g., the homeostatic microglia subset TAM1 reducing over time, or cDC2 and mDC increasing over time (Figure 2a). NK cells showed a statistically significant longitudinal increase from 1.26% ± 1.37 standard error of the mean (s.e.m) at 14 d to 5.21% ± 0.857 at 21 d to 8.44% ± 3.95 at 28 d (Figure 2a). To assess the transcriptional heterogeneity in NK cells across the p.i. time points, we conducted an unsupervised Multi-Omics Factor Analysis v2 (MOFA+) analysis of these cells followed by a pathway enrichment analysis using the enrichR algorithm [41,42]. MOFA+ infers a low-dimensional data representation and returns latent factors that account for variability in single-cell data [40]. Our analysis of the three p.i. time points returned eight latent factors (Figure 2b). Interestingly, Factor 1, which accounted for the most variability in the data, was mainly represented at 21 d and 28 d, while Factor 3 was mainly present at 14 d and 21 d, and Factor 5 had a gradual increase from 14 d to 28 d (Figure 2b). The enrichR-based analysis of the MSigDB Hallmark 2020 pathways showed myc-, E2F- and mitosis-related pathways among the top five pathways in Factor 1 (Figure 2c). Interestingly, Factor 3, which was downregulated at 28 d, contained interferon signaling among the top five pathways, while Factor 5 included hypoxia and glycolysis among its top five pathways (Figure 2c). In summary, an exploratory approach to profile immune cells during glioma progression showed a robust time-dependent increase in NK cells that was associated with a time-dependent downregulation of interferon signaling and upregulation of hypoxia pathways.

### 3.2. The Mouse Glioma Model GL261 Shows a Time-Dependent Accumulation of NK Cells with Downregulation of Activation Markers and Enhanced Lymphotoxin-β Expression

**Figure 3:**
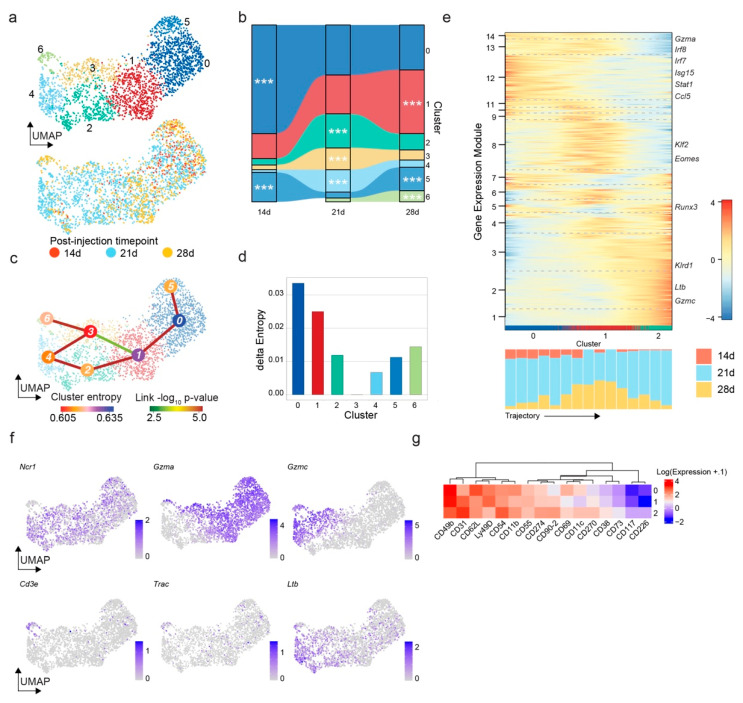
Characterization of NK cells during GL261 murine orthotopic glioma progression. (**a**) UMAP visualization of 2664 cells sub-clustered from the NK cell cluster of Figure 1c. The color coding of the top panel corresponds to the new cluster assignment. The bottom panel is color-coded by the p.i. time point. (**b**) Alluvial plot indicating the cluster distribution of the indicated p.i. time point. (**c**) UMAP visualization of the data from figure panel a superimposed by the lineage graph determined using the StemID algorithm. The nodes are color-coded by the transcriptional entropy of the indicated clusters; the link color indicates the *p*-value of the indicated connection. (**d**) Bar plot showing the transcriptional entropy differences across the cluster. Cluster 3 with the lowest entropy is set to 0. The color coding is consistent with the top part of figure panel a. (**e**) Heatmap of the locally smoothed gene expression z-scores along the trajectory between Clusters 0, 1 and 2. Genes with a correlation coefficient above 9 are summarized into gene expression modules; gene expression modules with at least 3 genes are shown. The bar plot below the heatmap indicates the time point composition of each step along the trajectory. (**f**) UMAP visualization of cells from figure panel a color-coded by the expression of the indicated genes. (**g**) Heatmap showing the log-transformed expression of the differentially expressed proteins in the indicated clusters.

Having identified NK cells as a highly variable immune cell type during glioma progression, we sought to characterize the transcriptional heterogeneity within each time point. To this end, we sub-clustered the NK cells and identified seven clusters (Figure 3a). A time-dependent analysis of the cluster composition at each time point showed a relative decline in Cluster 0 and a relative increase in Cluster 1 throughout glioma progression (Figure 3b). An enrichment analysis using hypergeometric testing showed statistically significant enrichment of Clusters 0 and 5 at 14 d, Clusters 2, 3 and 4 at 21 d and Clusters 1, 5 and 6 at 28 d. To assess stepwise gene expression changes, we applied the pseudotime analysis algorithm StemID [42]. This algorithm identified several trajectories (Figure 3c). We assessed the origin and direction of the trajectory based on the differences in transcriptional entropy. Following the notion that clusters with higher entropy represent transcriptional precursor states [51], we identified Cluster 0 as the earliest transcriptional state followed by Clusters 1 and 2 (Figure 3c,d). The heatmap visualization of the smoothed transcriptional changes along the identified trajectory showed a gradual downregulation of *Gzma*, the interferon signaling-associated genes *Irf7*, *Isg15* and *Stat1* and the chemokine gene Ccl5 with a concomitant upregulation of *Gzmc* and *Ltb* (Figure 3e). Alterations of interferon genes and *Ccl5* expression were previously associated with NK cell exhaustion [38,52]. The TNF superfamily member gene LTB has pleiotropic functions, including cancer inflammation, tertiary lymphoid structure and NK cell development [33,34,35,53]. A visualization of the stepwise cluster composition of the trajectory showed that the second half of the trajectory was almost exclusively composed of cells from 21 d and 28 d (Figure 3e). Representative gene expression UMAP plots confirmed the gene expression patterns of *Gzma*, Gzmc and *Ltb* along the trajectory (Figure 3f). Cells from clusters other than Cluster 6 showed expression of the NK marker *Ncr1*/Nkp46, while Cluster 6 showed expression of T cell markers, including *Cd3e* and *Trac*. These cells may represent NKT cells or a recently identified subset of glioma-associated T cells expressing NK cell markers [26]. The enrichment of *Cd3e* and *Trac* in cluster 6 suggests that the remaining clusters consist of bona fide NK cells. The gene ontology analysis indicated that Cluster 0’s cells were associated with the terms accompanying iron homeostasis, commonly upregulated in the hypoxic tumor environment [54] (Supplementary Figure S1a). Cluster 1 showed expression of inflammation and extracellular matrix-associated terms, and Cluster 2 was associated with protein and RNA homeostasis-related terms (Supplementary Figure S1a). To identify which gene regulatory elements were driving the identified transcriptional changes, we conducted a SCENIC analysis [45]. Interestingly, we identified a downregulation of type I (IRF7) and type II interferon (STAT1) and NF-κB (KLF2) signaling (Supplementary Figure S1b). Finally, protein validation based on CITE-Seq data from 21 d cells [20] showed a downregulation of several NK cell activation markers (Ly49D, CD69) along the trajectory between Clusters 0, 1 and 2 (Figure 3g). Interestingly, Cluster 2 showed that upregulations of CD117/c-kit and LTBR signaling have been implicated in lymphoid tissue development and tertiary lymphoid structures during development and in cancer [53,55]. The upregulation of CD117/c-kit and LTB suggest an NK cell subset with a dysfunctional/de-differentiated transcriptional state [34]. In summary, we identified an NK cell subset associated with glioma progression. These cells showed a downregulation of NK activation markers and interferon signaling-associated genes, suggesting a dysfunctional NK transcriptional cell state.

### 3.3. Myeloid Cells Are the Main Putative Ligands for Lymphotoxin-β

LTB signaling occurs via a membrane-bound (LTα_1_LTβ_2_) heterotrimer binding to LTBR [33]. While LTB is mainly expressed on lymphoid cells, LTBR is found on stromal and myeloid cells. We utilized the cell–cell interaction analysis algorithm CellphoneDB to explore this crosstalk across immune cell subsets [56]. The analysis suggested several putative interactions across the immune cell types at the available p.i. time points (Supplementary Figure S2a). We found several T cell subsets involved in putative LTB–LTBR crosstalk (Supplementary Figure S2b). Interestingly, NK cells and cDC2 showed higher expression of LTB between 14 d and 21 d, while neutrophils showed a downregulation of LTB between these time points. *Lta* was expressed by regulatory and effector T cells with the mean *Lta* expression increasing between 14 d and 21 d (Supplementary Figure S2c). The 28 d day time point was underrepresented in this analysis, which may be due to the technical differences of this dataset, as the number of the identified interactions in this dataset overall is lower (Supplementary Figure S2a). In summary, during glioma progression, NK cells appear to be increasingly involved in LTB-mediated crosstalk with myeloid cells and likely other non-immune cells that are not present in this dataset.

### 3.4. Human Glioblastoma Progression Is Associated with Dysfunctional NK Cell Accumulation and Enhanced Lymphotoxin-β Expression

**Figure 4:**
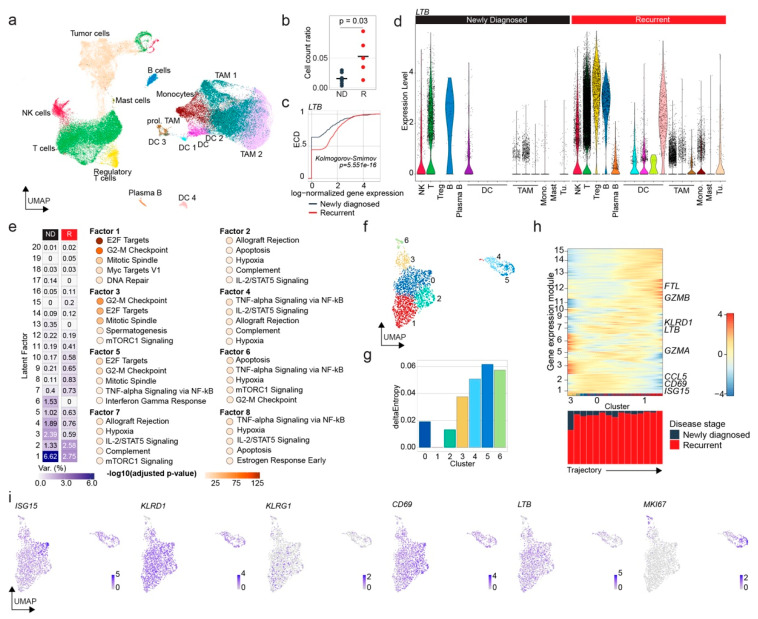
Dysfunctional NK cells accumulate in recurrent human glioblastoma. (**a**) UMAP of 61,164 human glioblastoma-associated cells from the Antunes et al. dataset. The color coding shows immune cell assignment as provided by the authors of the study [20]. The tumor cells were assigned based on the expression of glioblastoma-associated gene signatures. Non-neoplastic oligodendrocytes and immune cells that were not assigned by the authors were excluded. (**b**) Dot plot visualization of the NK cell count relative to all immune cells color-coded by the tumor stage. The indicated *p*-value was calculated using an unpaired two-sided Wilcoxon rank-sum test. (**c**) Empirical cumulative density function plot of the *LTB* expression in NK cells color-coded by the tumor stage. The indicated *p*-value was calculated using a two-sided two-sample Kolmogorov–Smirnov test. (**d**) Dot violin plots showing *LTB* expression in the respective cell types in ND and R glioblastomas from figure panel a. (**e**) Heatmap visualization of latent factors determined by MOFA+ analysis. The color scale indicates the percentage of variance explained. For enhanced readability, the respective values are indicated in each cell. The enrichR-based pathway enrichment analysis of MSigDB Hallmark 2020 pathways. The color of the dots indicates the -log10-transformed adjusted *p*-values of the enrichment. (**f**) UMAP visualization of 2,158 cells sub-clustered from the NK cell cluster of figure panel a. The color coding of the top panel corresponds to the new cluster assignment. The bottom panel is color-coded by the p.i. time point. (**g**) Bar plot showing the transcriptional entropy differences across the cluster. Cluster 1, with the lowest entropy, is set to 0. The color coding is consistent with that of figure panel f. (**h**) Heatmap of the locally smoothed gene expression z-scores along the trajectory between Clusters 3, 0 and 1. Genes with a correlation coefficient above 9 are summarized into gene expression modules; gene expression modules with at least 3 genes are shown. The bar plot below the heatmap indicates the tumor stage composition of each step along the trajectory. (**i**) UMAP visualization of cells from figure panel f color-coded by the expression of the indicated genes.

To validate our findings in humans, we turned to a dataset of immune cells FACS-sorted from newly diagnosed (ND) and recurrent (R) glioblastomas [20]. This dataset contained tumor cells along with diverse immune cell populations (Figure 4a). Notably, we found a significantly higher relative numeric abundance of NK cells in R glioblastomas compared to ND ones (Figure 4b). Apart from higher numeric abundance, cells from R glioblastomas showed significantly higher transcript abundances of LTB compared to their ND glioblastoma-associated counterparts (Figure 4c). Likewise, LTB expression across the immune cell types was enhanced in R glioblastomas (Figure 4d). Next, we conducted a MOFA+ analysis of the latent factors underlying the transcriptional phenotypes of ND- and R-glioblastoma-associated NK cells followed by an enrichR-based pathway enrichment analysis. We found a downregulation of the factors associated with the cell cycle (Factors 1, 3, 5, 6) and interferon-gamma signaling (Factor 5) and an upregulation of the factors associated with apoptosis (Factors 2 and 8) (Figure 4e). Next, we sub-clustered the NK cells to assess the stepwise transcriptional changes (Figure 4f). StemID suggested a trajectory based on transcriptional similarity and entropy from Cluster 3 over Clusters 0 to 1 (Figure 4g). Clusters 0 and 1 almost exclusively consist of R-glioblastoma-associated NK cells (Figure 4h). Along the trajectory, we found a downregulation of *ISG15*, *CD69* and *CCL5* and an upregulation of *LTB*, *GZMB* and *FTL* (Figure 4h,i). Interestingly, we found an upregulation of *KLRG1*, a marker gene for exhausted NK cells [36,37] (Figure 4i). A gene ontology analysis showed enrichment of the cytokine and chemokine function-associated terms in Cluster 0 and the RNA and protein turnover-associated terms in Cluster 1 (Supplementary Figure S3a). The SCENIC analysis identified a reduction in gene expression under the control of *STAT1*, *IRF1* and *IRF7* between Clusters 0 and 1 (Supplementary Figure S3b). In summary, single-cell RNA-sequencing data from human ND and R glioblastomas showed an accumulation of NK cells with reduced expression of activation, interferon-gamma and cell cycle genes and enhanced expression LTB and apoptosis-associated genes, suggesting an exhausted NK cell phenotype. NK cell exhaustion has been associated with the loss of cell proliferation markers, reduced interferon signaling and apoptosis signaling [38]. Strikingly, the data showed broad similarities with the mouse data above.

### 3.5. LTB–LTBR Crosstalk Is Associated with MES-like Regions of Glioblastomas

**Figure 5:**
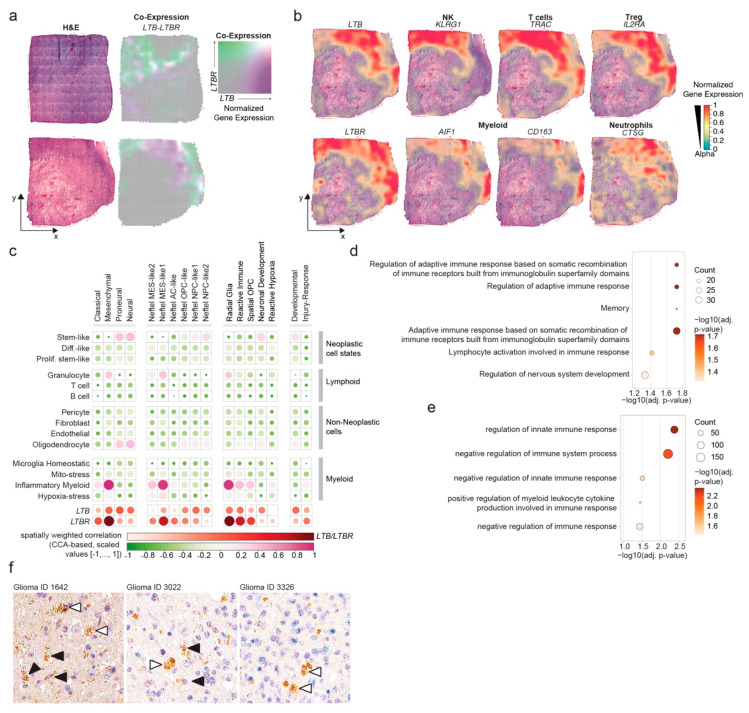
LTB expression is spatially associated with lymphoid cell and neurodevelopmental genes and clinically associated with poor prognosis. (**a**) H&E images of two independent glioblastoma samples analyzed using 10X Visium from a published dataset [10]. Spatial co-expression of *LTB* and *LTBR* is color-coded. (**b**) The normalized spatial gene expression of the indicated genes and the cell types that these genes are enriched in is superimposed with the bottom case from figure panel a. (**c**) Dot plot visualizing the spatially weighted correlation analysis between the expression of the *LTB* and *LTBR* with glioblastoma-associated gene sets. (**d**) Dot plot indicating the gene ontology terms enriched in dots with *LTB* expression. (**e**) Dot plot indicating the immune-associated gene ontology terms enriched in dots with *LTBR* expression. (**f**) Representative images of LTBR immunohistochemistry from the Human Protein Atlas. The sample IDs are presented above the images. Filled arrowheads indicate ramified microglia-like cells; empty arrowheads indicate cells with foamy macrophage-like morphology.

Next, we utilized a spatial transcriptomics dataset of human glioblastomas to map *LTB* and *LTBR* expression in situ [10]. We found that transcripts of *LTB* and its receptor *LTBR* are found in adjacent tumor regions with overlaps at the intercept of these regions (Figure 5a). The spatial transcript dataset confirmed a correlation of *LTB* transcripts with the NK exhaustion marker gene *KLRG1* and lymphoid cell markers, including *TRAC* and *IL2RA*, while *LTBR* transcripts were associated with the pan-myeloid marker AIF1/Iba-1, the macrophage marker *CD163* and the neutrophil marker *CTSG* (Figure 5b). Spatially weighted correlation analysis for *LTB* showed a strong association with MES-like glioblastoma regions with reactive gene signatures and regions showing developmental gene expression patterns, such as the OPC- and NPC-like regions (Figure 5c). LTBR expression was spatially correlated to MES-like regions with myeloid and reactive gene expression programs (Figure 5c). These findings were further confirmed by an unbiased gene ontology term enrichment analysis of differentially expressed genes in spatial dots expressing *LTB* and *LTBR*, respectively. *LTB*-expressing dots showed enrichment of adaptive immunity and nervous system development terms (Figure 5d). *LTBR*-expressing dots were characterized by the enrichment of innate immune-related gene ontology terms (Figure 5e). In situ protein validation from glioma samples of the Human Protein Atlas [57] confirmed the expression of LTBR protein in different samples in cells that had the morphological features of ramified microglia cells and foamy macrophages (Figure 5f). Its ligand LTB was undetectable in glioblastoma sections due potentially to the challenges of detecting secreted proteins in immunohistochemistry. Next, we characterized the expression of *LTB* and *LTBR* in the TCGA and CGGA datasets [58,59]. The cancer transcriptional subtype assignment [4,60] was summarized elsewhere [61]. Both datasets consisted mostly of AC-like and MES-like glioblastomas (Supplementary Figure S4a). In line with the spatial gene expression data, *LTBR* expression was highest in MES, followed by classical (CL) samples; *LTB* expression was highest in MES, followed by Proneural (PN) samples (Supplementary Figure S4b). To assess the impact of *LTB* expression on the clinical outcome of glioblastomas, we compared overall survival in low- and high-LTB expressing samples of both datasets with the median as the cutoff. High *LTB* expression appeared to be associated with a reduced overall survival (Supplementary Figure S4c), whereas *LTBR* expression did not reach statistical significance. In summary, we found that *LTB* expression was spatially correlated with the expression of NK and T cell gene expression. It was mainly found in MES-like and neuroectodermal progenitor-like regions and appeared to be associated with clinical prognosis.

## 4. Discussion

Glioblastomas are aggressive brain tumors that remain incurable. As more than 50% of a glioblastoma tumor’s mass consists of myeloid cells alone [62], understanding the immune compartment of the glioblastoma microenvironment may hold the key to targeted therapies for this tumor entity. In the present study, we have conducted an integrated analysis of glioma-associated immune cells during glioma progression in humans and mice. We found that NK cells show a time-dependent accumulation during the progression of murine orthotopic GL261 gliomas. Transcriptionally, NK cells adopt a dysfunctional phenotype with a downregulation of interferon genes and stimulatory receptor proteins, including CD69 and LY49D. At the same time, these cells upregulate the LTB gene, a TNF superfamily member associated with lymph organ development. In human glioblastoma tissues, LTB expression was spatially correlated with the MES-like subtype, and neurodevelopmental gene expression signatures appeared to be associated with prognosis.

The glioblastoma immune microenvironment has been subject to extensive research in recent years [14]. While a special focus has been directed at macrophages as the most common immune cells of glioblastoma [62], lymphoid and dendritic cells have been studied extensively for targeted immune therapies, including immune checkpoint inhibition, vaccines and chimeric antigen receptor (CAR) T cell therapies [63,64,65,66,67]. As immune checkpoint inhibitors have shown moderate clinical efficacy, the molecular and cellular mechanisms of glioblastoma-associated immunosuppression are under continued investigation. Interestingly, autologous tumor-infiltrating T and NK cells were shown to regain tumor lysis activity after ex vivo stimulation, underscoring the strong immunosuppressive influence of glioblastomas [68,69]. By overcoming glioblastoma-mediated immune suppression, immunocytokines have recently shown impressive results in inducing inflammation and T cell-mediated tumor cell lysis in glioblastomas in vivo [70]. Other promising approaches include CAR NK cells [71,72]. In summary, while various therapeutic strategies are being developed, the molecular mechanisms of glioblastoma-associated immune suppression and tumor cell infiltration are gradually being understood.

Accumulating evidence points toward glioblastomas co-opting neurodevelopmental programs for tumor progression. One insidious feature of this co-option is the integration of tumor cells into existing neural cell networks that drive tumor progression [6,7,8]. The immune system adapts to the de-differentiation tendency of tumors by producing immature immunosuppressive cells [73]. In this context, LTB may serve as a molecular marker of “immune de-differentiation”. Its role in lymphoid organ development and NK cell maturation is well characterized [33,34]. Interestingly, fetal lymphoid tissue inducer cells are NK cell-like cells that are also found postnatally and lack interferon-γ expression [74]. A chronically inflammatory milieu in glioblastoma drives profound changes in the immune compartment [20,21,22,23]. During chronic inflammation, LTB secretion induces the formation of tertiary lymphoid structures, the ectopic lymphoid cell aggregates [75]. NK cells are not the only cells that express LTB. It is also expressed by B and T lymphoid cells, tumor-associated macrophages and monocytes. Interestingly, a recent study highlighted an NK-like phenotype in T cells showing enhanced LTB expression [26]. Some degree of transcriptional convergence in glioblastoma has been reported within the myeloid cell compartment [20,21,23] (e.g., between microglia, CNS-associated macrophages and monocyte-derived macrophages) and even between myeloid and tumor cells [16]. These observations may support the notion of glioma-associated immune de-differentiation across different cell lineages.

The present study has outlined some similarities and crucial differences in the observed NK cell transcriptional phenotype across human and murine gliomas. In both systems, we find an accumulation of NK cells with enhanced expression of LTB and a downregulation of activation markers. In humans, enhanced LTB expression was primarily associated with MES-like tumor transcriptional subtypes that show strong transcriptional similarities with the GL261 cell line [76]. Differently, we found that human NK cells show a higher expression of the NK exhaustion marker gene KLRG1 and apoptosis-associated genes [38]. This may occur due to the overall shorter observational time frames between murine gliomas (weeks) and human glioblastomas (months). Other aspects determining interspecies differences are interindividual genetic and lifestyle variations, microbiota and therapeutic interventions in the patient cohort. In summary, we found a robust cross-species NK cell transcriptional signature associated with glioma progression.

This study has several limitations that need to be considered. The murine and human data were obtained in different laboratories, leading potentially to batch effects. As a meta-analysis, the nature of this study is observational; hence, it is lacking mechanistic corroboration. To obtain insights into the functional NK cell states beyond transcriptomics, in vitro analyses are required. Furthermore, protein validation of the main marker genes across humans and mice is advisable. We have strived to address these and other limitations by using state-of-the-art single-cell RNA sequencing data integration algorithms and analyzing these data using distinct, complementary analytical pipelines. Finally, protein validation for the human tissues was achieved using a public protein expression database.

## 5. Conclusions

Here, using human and murine glioma data, we have shown a distinct NK cell transcriptional phenotype associated with glioma progression. These cells are characterized by reduced expression of activation markers and enhanced LTB gene expression. High LTB gene expression was mainly associated with MES-like and neurodevelopmental glioblastoma subtypes. The novelty of our study is the description of a previously underappreciated NK cell state that may underlie the previously reported NK cell dysfunction [69]. Our findings contribute to the understanding of the immune component of the glioblastoma microenvironment and may help facilitate the development of immune therapies for this tumor.

## Data Availability

The count data for this project are available under GSE136001, GSE166218 and https://www.brainimmuneatlas.org/download.php (accessed on 15 August 2022). Bulk RNA-Seq data were downloaded from http://cgga.org.cn/download.jsp (accessed on 15 August 2022) and https://www.proteinatlas.org/about/download (accessed on 15 August 2022). The images for Figure 5f can be found under the indicated IDs at the following URL: https://www.proteinatlas.org/ENSG00000111321-LTBR/pathology/glioma#img (accessed on 15 August 2022).

## Supplementary Materials

Supporting information can be downloaded at https://www.mdpi.com/article/10.3390/cancers14194915/s1, including Figure S1: Gene ontology and regulatory element analysis of murine GL261-associated NK cells; Figure S2: Cell–cell interactions of the immune cells in the datasets suggest lymphoid-to-myeloid cell communication in lymphotoxin signaling; Figure S3: Gene ontology and regulatory element analysis of human glioblastoma-associated NK cells; Figure S4: Validation of findings using TCGA and TGGA glioblastoma datasets [77].
