# Supplementary material for: Transcriptome Analysis Identifies Accumulation of Natural Killer Cells with Enhanced Lymphotoxin-β Expression during Glioblastoma Progression
Source: Cancers (Basel). 2022 Oct 7;14(19):4915. doi: 10.3390/cancers14194915 (PMC9563981; doi:10.3390/cancers14194915)
Supplement: Supplementary file 1 [file cancers-14-04915-s001.zip › cancers-1937739-supplementary.pdf]

cancers

MDPI

Supplementary Materials: Transcriptome Analysis Identifies Accumulation of Natural Killer Cells with Enhanced Lymphotoxin-β Expression during Glioblastoma Progression

Gianni Monaco, Ashkan Khavaran, Adrià Dalmau Gasull, Jonathan Cahueau, Martin Diebold, Chintan Chhatbar, Mirco Friedrich, Dieter Henrik Heiland and Roman Sankowski

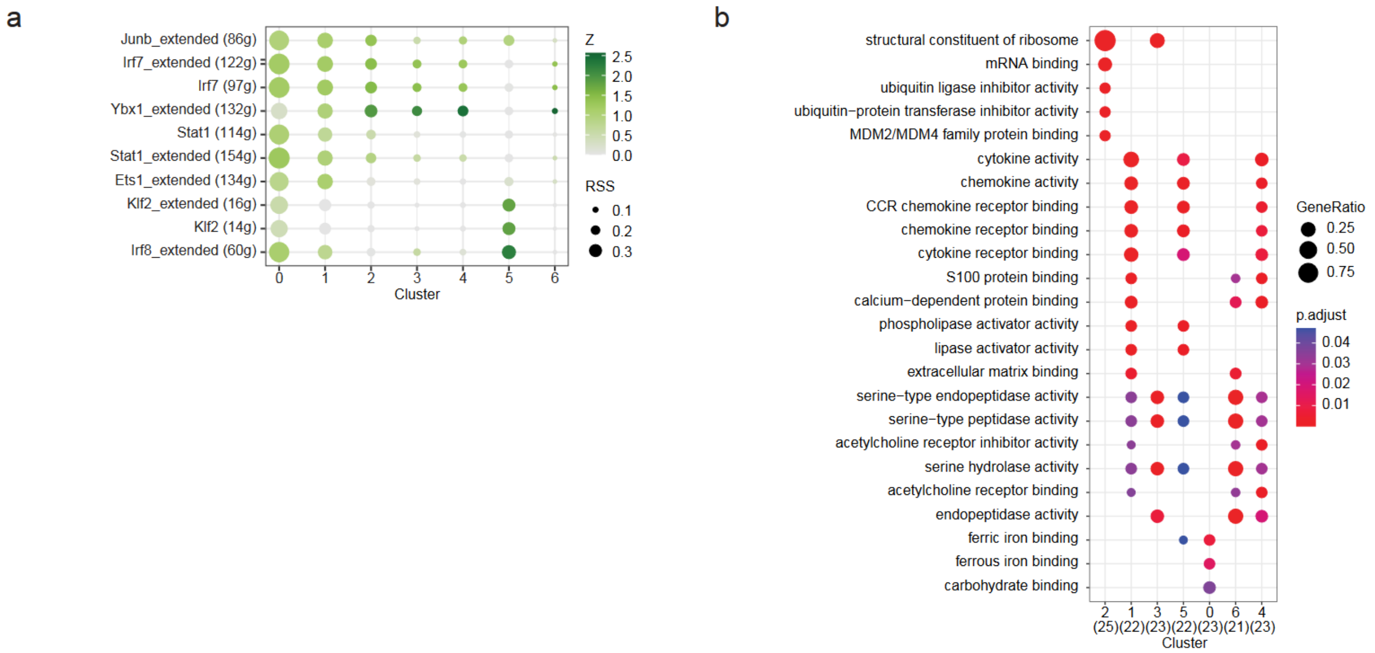

Figure S1. Gene ontology and regulatory element analysis of murine Gl261-associated NK cells. (**a**) Geneontology enrichment analysis based on the expression of the top 25 differentially expressed genes in each cluster. The dotsize indicates the number of genes present in the cluster, the color-coding represents the adjusted *p*-value. (**b**) Dot plot showing the gene regulatory elements across clusters identified by the SCENIC algorithm. The color-coding indicatesz-score as the scaled regulon activity score and the dot size indicates the regulon specificity score [1] quantified by the Jensen-Shannon divergence of the respective cluster.

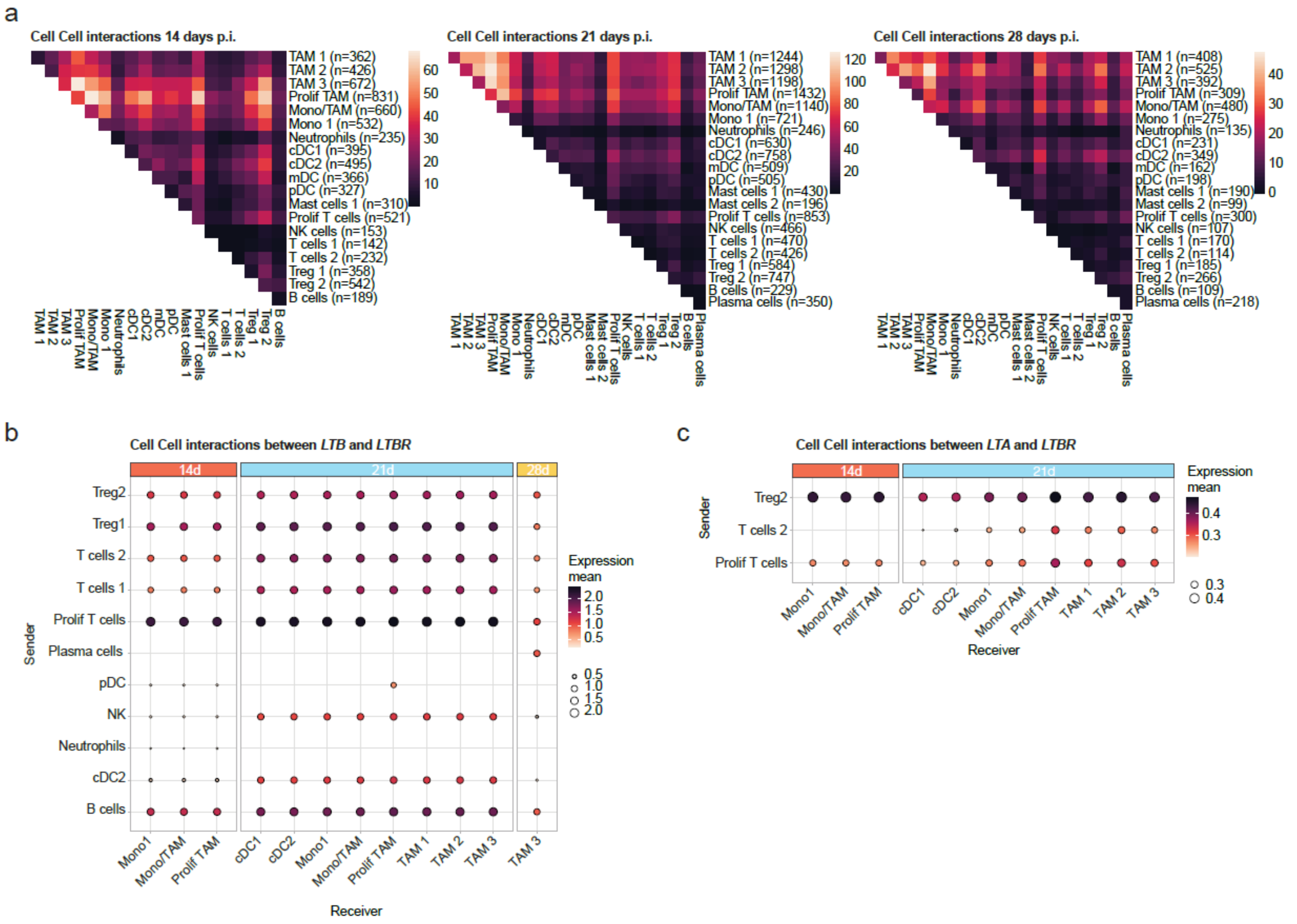

Figure 2. Cell-cell interactions of the immune cells in the datasets suggests lymphoid-to-myeloid cell communication in lymphotoxin signaling. (**a**) Statistically significant putative cell-cell interaction counts suggested by Cellphone DB between the respective datasets. (**b**) Dot plot depicting the mean expression of the interacting genes LTB and LTBR across the immune cells present in the datasets. The x-axis depicts the receiver cell types expressing LTBR, the y-axis the sender cell types expressing LTB. The dot size and color are encoding the mean expression of the interacting genes. (**c**) Dot plot depicting the mean expression of the interacting genes LTA and LTBR across the immune cells present in the datasets. The x-axis depicts the receiver cell types expressing LTBR, the y-axis the sender cell types expressing LTA. The dot size and color are encoding the mean expression of the interacting genes.

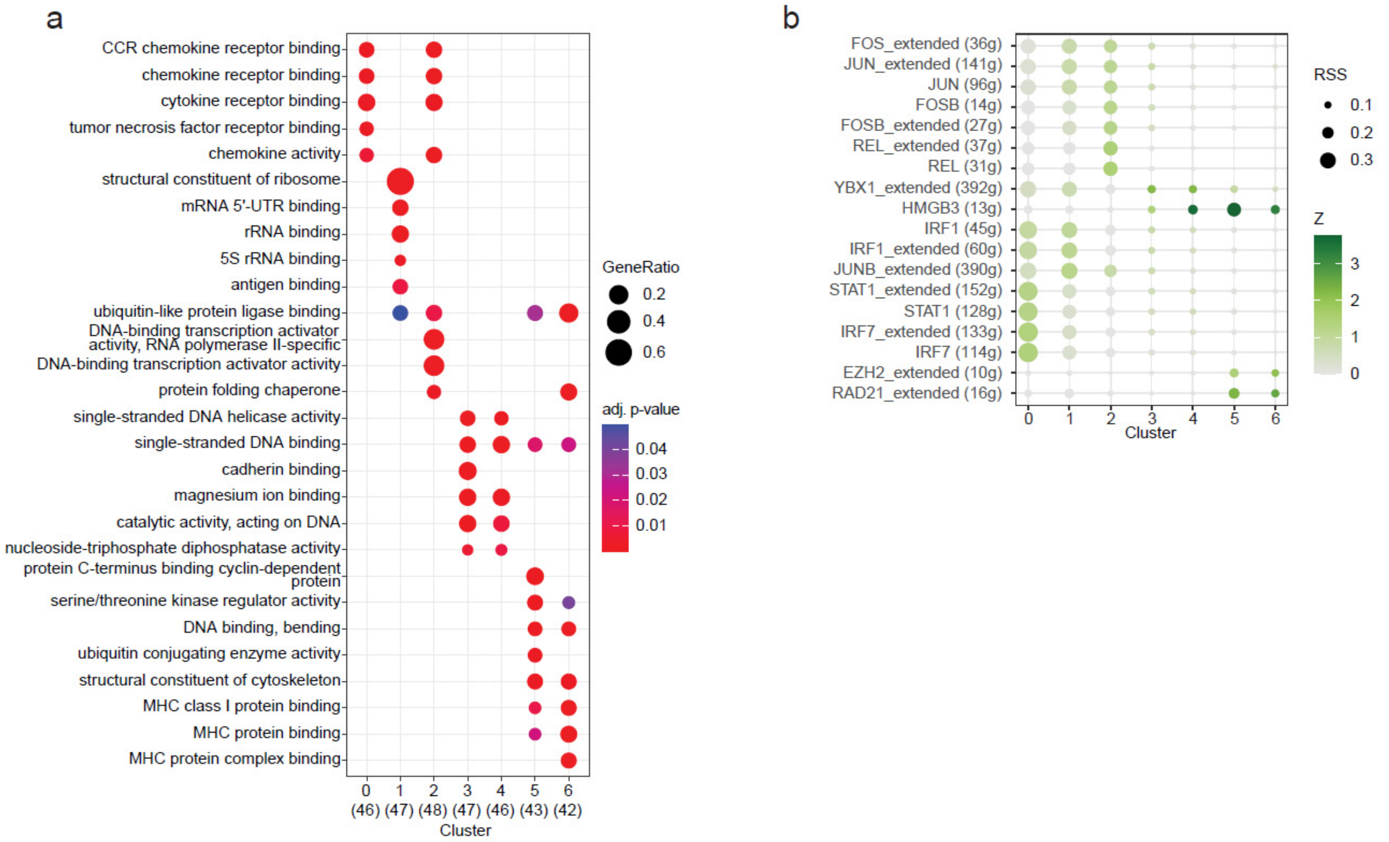

Figure 3. Gene ontology and regulatory element analysis of human glioblastoma-associated NK cells. (**a**) Gene ontology enrichment analysis based on the expression of the top 25 differentially expressed genes in each cluster. The dot size indicates the number of genes present in the cluster, the color-coding represents the adjusted *p*-value. (**b**) Dot plot showing the gene regulatory elements across clusters identified by the SCENIC algorithm. The color-coding indicates z-score as the scaled regulon activity score and the dot size indicates the regulon specificity score [1] quantified by the Jensen-Shannon divergence of the respective cluster.

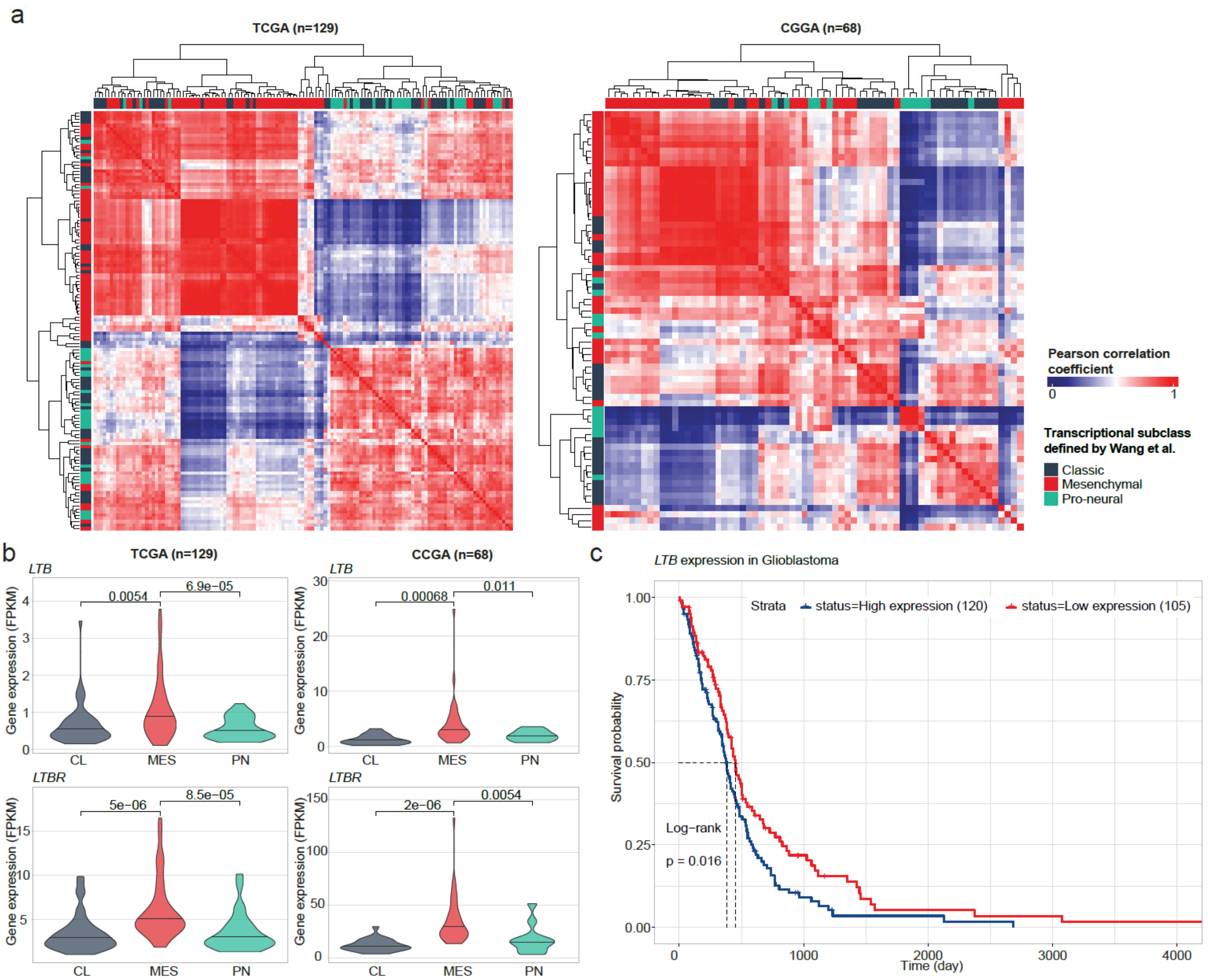

Figure 4. Validation of findings using TCGA and TGGA glioblastoma datasets. (**a**) Correlation heatmap of TCGA and CGGA samples with externally assigned transcriptional subtypes. The color bar indicates the Pearson correlation coefficient. Sample clustering was achieved using the Euclidean distance metric and complete clustering method. Only glioblastoma samples with available subtype classification were included. (**b**) Violin plots visualizing the gene expression of LTB and LTBR in Fragments per kilobase of transcript per million mapped fragments (FPKM) across the transcriptional subtypes in the TCGA and CGGA cohorts. The p-values were obtained from unpaired unpaired two-sided Wilcoxon rank sum tests. (**c**) Kaplan-Meier curves of high and low expressors of LTB from the glioblastoma samples in the TCGA and CGGA cohorts. The *p*-value was calculated using the Log-rank test.

Reference

1. Suo, S.; Zhu, Q.; Saadatpour, A.; Fei, L.; Guo, G.; Yuan, G.-C. Revealing the Critical Regulators of Cell Identity in the Mouse Cell Atlas. *Cell Rep.* **2018**, *25*, 1436–1445.e3. https://doi.org/10.1016/j.celrep.2018.10.045.
